# A multicentre evaluation of cardiovascular risk prediction tools in people living with HIV across England (ECARTH study)

**DOI:** 10.1111/hiv.70282

**Published:** 2026-07-20

**Authors:** Ming Jie Lee, Ashley Murray, Colette Smith, Zakee Abdi, Alastair Duncan, Ellen Dwyer, Mark Gompels, Merle Henderson, Margaret Johnson, Frances Lander, Gavin Manmathan, Jonathan Mok, Nnenna Ngwu, Coral Pratt, Iain Reeves, Razan Saman, Alice Sharp, Eleanor Taylor, Jhia Jiat Teh, Richard Stevens, Thomas R. Fanshawe, Jienchi Dorward, Ranjababu Kulasegaram, Anthony S. Wierzbicki

**Affiliations:** ^1^ Department of Infectious Disease Imperial College London London UK; ^2^ Harrison Wing, Department of HIV Guy's and St Thomas Hospitals NHS FT London UK; ^3^ Canberra Sexual Health Centre Canberra Australian Capital Territory Australia; ^4^ Nuffield Department of Primary Care Health Sciences University of Oxford Oxford UK; ^5^ Institute of Global Health University College London London UK; ^6^ Royal Free London NHS FT London UK; ^7^ West Middlesex University Hospital, Chelsea and Westminster Hospital NHS FT London UK; ^8^ North Bristol NHS Trust Bristol UK; ^9^ Imperial College Healthcare NHS Trust London UK; ^10^ Homerton University Hospital NHS FT London UK; ^11^ Leeds Teaching Hospital NHS Trust Leeds UK; ^12^ Centre for the AIDS Programme of Research in South Africa (CAPRISA) University of KwaZulu‐Natal Durban South Africa; ^13^ Department of Metabolic Medicine/Chemical Pathology Guy's and St Thomas Hospitals NHS FT London UK

**Keywords:** cardiovascular disease, heart disease risk factors, HIV, myocardial Ischaemia, stroke

## Abstract

**Introduction:**

It remains unclear which cardiovascular disease (CVD) risk scores are optimized for people living with treated HIV with high rates of viral suppression. We evaluated the performance of commonly used CVD risk scores in a population of primarily well‐treated HIV patients in England.

**Methods:**

A multi‐centre retrospective cohort analysis was performed, including people living with HIV (PLWH) above the age of 40 with no prior major adverse CVD events (MACE), regularly attending 7 HIV clinics in England during 2014. Outcomes were MACE over the following 5 years: myocardial infarction, invasive cardiac procedure e.g. primary angioplasty, cerebrovascular events, new heart failure, and CVD‐related death. Clinical outcomes were aligned with the respective risk scores. The following CVD risk models were evaluated: the UK primary‐care validated QRISK3, Framingham laboratory and non‐laboratory (Office) scores, Data collection on Adverse events of anti‐HIV Drugs (D:A:D), Pooled Cohort Equation (PCE), and the World Health Organization (WHO) laboratory and non‐laboratory models. Models were scaled for 5‐year CVD estimates. We assessed the discrimination and calibration of these 5‐year models within our population. Multiple imputation was used to address missing data.

**Results:**

Of 2582 people, 94 had at least one MACE documented during follow‐up. All seven scores demonstrated moderate discrimination. QRISK3 demonstrated the best calibration in this cohort with an O:E ratio of 1.169, 95% CI 0.950, 1.439, mean calibration‐in‐the‐large (CITL) value of 0.156 (95% CI −0.052, 0.364) and slope of 0.897 (0.682, 1.113). Framingham Office generally overpredicted risk, while WHO scores and D:A:D generally underpredicted risk. Imputing for missing data yielded results similar to the complete case analysis.

**Conclusions:**

CVD risk scores demonstrated moderate performance in a well‐treated PLWH cohort. Our findings emphasize the need to calibrate each CVD risk score to the local population to guide prioritization of clinical resources for CVD prevention.

**Evidence in Context:**

Evidence before this study: We systematically searched Medline, Embase, and Cochrane Library database from January 1995 to August 2023, using the following terms ‘HIV/AIDS and Cardiovascular Disease (CVD) outcomes’, with an updated search to 2025. The risk of CVD has been reported to be up to two times that of people living without HIV, along with increased rates of heart failure and sudden death. Commonly used CVD risk scores have demonstrated variable performance for PLWH, and only the D:A:D study equation has been specifically validated in an HIV‐positive population. Within an era of modern ART regimes and high rates of viral suppression, there have been numerous comparisons of CVD risk across scoring systems, but few studies with observed outcomes of CVD rates to validate the predictions. It remains unclear which CVD risk calculator is the most suitable for PLWH. The use of routinely collected clinical data may also impact the accuracy of results due to the presence of incomplete or missing data fields. In this study, we aimed to evaluate the performance of commonly used CVD risk scores in a population of primarily well‐treated HIV patients in England using routinely collected clinical data. Added value of this study: We demonstrate that of the seven CVD risk scores evaluated (QRISK3, Framingham laboratory and non‐laboratory (Office) scores, D:A:D, Pooled Cohort Equation (PCE), and the WHO laboratory and non‐laboratory models), all showed moderate discrimination compared to observed major adverse cardiovascular events (MACE) rates in an English multicentre cohort of people living with HIV attending clinics. QRISK3, using a large UK primary care database to validate predicted CVD risk, appeared to be most closely calibrated to CVD outcomes in our cohort without the need for additional calibration. Our findings also suggest that the use of non‐laboratory scores, which have been suggested for low and middle‐income settings where laboratory results may not be available, should be interpreted with caution, and further testing may be required due to their miscalibration in this cohort. Implications of all the available evidence: Using real‐world clinical data from people with HIV who are engaged in care in England, these findings emphasize the need to calibrate each CVD risk score to the local population it is used for in order to accurately guide prioritization of clinical resources for primary CVD prevention.

## INTRODUCTION

Progress in highly effective antiretroviral therapy (ART) has led to increased life expectancy in people living with HIV (PLWH) approaching that of the general population. However, persisting disparities in co‐morbidities‐free survival, particularly non‐infectious conditions including cardiovascular disease (CVD), remain for PLWH [1, 2]. The risk of myocardial infarctions and strokes for PLWH has been reported to be up to two times that of people living without HIV, along with increased rates of heart failure and sudden death [3, 4, 5].

This increased risk is likely driven by multiple factors. Amongst PLWH, traditional CVD risk factors and co‐morbidities such as smoking, dyslipidaemia, hypertension and diabetes are more prevalent [6, 7, 8, 9]; HIV‐associated chronic inflammation and immune activation also play a major role [10, 11, 12]. In addition, antiretroviral drug classes can adversely impact CVD risk profiles [13, 14, 15].

Recently, a large multinational randomized controlled trial of daily low‐dose pitavastatin for primary prevention of CVD in PLWH with low‐to‐moderate risk demonstrated a 35% relative hazard risk reduction in major CVD events [16]. These findings have already impacted clinical practice on recommending statin use in PLWH aged over 40, regardless of CVD risk in the UK [17] and the USA [18]. Guidelines in the general population recommend the use of CVD risk calculators as the best method to identify individuals at high risk of future events. Accurate CVD risk estimation informs both the prioritization of people at the highest risk of CVD when focusing resources on counselling on CVD risk prevention and allows individuals to make informed decisions when weighing the risks and benefits of starting statin or other lipid‐lowering therapies for primary prevention.

Cardiovascular disease risk models have been recommended for clinicians to assess CVD risk in PLWH [19, 20, 21]. There is evidence that these scores underestimate the true CVD risk in PLWH [4, 19]. Only the Data collection on Adverse events of anti‐HIV Drugs (D:A:D) study equation has been specifically validated in a HIV‐positive population, with the majority of individuals not effectively virally suppressed on ART [20]. In the era of modern ART regimes and high rates of viral suppression with modern ART implementation guidelines, previous estimates of the burden of chronic HIV infection and its contribution to calculated CVD risk should be re‐evaluated. It is unclear which CVD risk calculator is most suitable for the modern cohort of PLWH. Previous head‐to‐head comparisons of risk scores found that a higher overall CVD risk was attributed to PLWH when using the Framingham Heart Study (FRS) scores compared to D:A:D and the US atherosclerotic cardiovascular disease (ASCVD) models; however, a limitation was heterogeneity of outcomes included in each model [21].

In this multicentre, retrospective cohort analysis, we compared estimated 5‐year rates of CVD events using commonly used CVD risk models, with actual observed events in a 5‐year follow‐up period of a representative population of PLWH attending HIV clinics across England. In addition to comparing overall event estimates, we evaluated how well each model differentiated between individuals who did and did not develop cardiovascular events, and whether the absolute risks predicted by each model accurately reflected real‐world outcomes in the contemporary ART era.

## METHODS

### ECARTH study design and cohort

The Evaluation of Cardiovascular Risk Tools in PLWH (ECARTH) study is a multi‐centre retrospective cohort analysis which comprised either whole clinic cohorts or randomly selected cohorts of 50 or a 100 PLWH within individual clinics above the age of 40 with no prior history of CVD events, regularly attending seven HIV centres in England during 2014 (Table S1). Centres included in the study served diverse patient cohorts across the country, ranging from inner city to regional populations, and the random selection of clinical cohorts allowed participating centres to contribute data in accordance with a commonly used British HIV Association audit protocol [22]. This cohort reflected the real‐world routinely collected data when people attended clinics for their HIV care. PLWH with a history of any CVD events were excluded from analysis, including any confirmed diagnosis of myocardial infarction with or without ST‐elevation, previously documented to have undergone invasive cardiac procedures, or any diagnosis of any cerebrovascular events, including transient ischaemic attacks.

The study was approved by the UK Health Research Authority (UK HRA) (REC reference 20/HRA/6306) and deemed exempt from Research Ethics Committee review due to the retrospective nature of the study and review of previously collected, non‐identifiable routine clinical information.

### Data collection

Data from electronic patient records documented at routine clinical visits were recorded at individual sites by trained investigators from the team directly involved in their care. Data extracted included baseline demographics, HIV‐care and medications‐related characteristics, dates of clinic visits, co‐morbidities and concomitant medications. The CVD outcomes in the 5 years following the initial clinic visit were also recorded. Data were pseudonymized at each contributor site prior to grouping and statistical analysis.

### Definitions

Relevant major adverse CVD events (MACE) were defined as documented myocardial infarction (MI) or invasive cardiac procedure (ICP), e.g., primary angioplasty, cerebrovascular events, new diagnosis of heart failure (for FRS only), and CVD‐related death. Myocardial infarctions include both ST elevation and non‐ST elevation myocardial infarcts. Clinical outcomes were aligned with the respective risk scores.

The initial clinic visit was defined as the first clinic visit in 2014 with the most detailed information available. The follow‐up period was initiated from the date of the initial visit and censored at the last clinic visit within 5 years or 5 years from the date of the initial visit (whichever occurred first). Baseline investigation results were taken as the last result available prior to the initial visit. Steroid use was defined as a regular dose of daily corticosteroids greater than or equivalent to 5 mg prednisolone once a day. Anti‐diabetic agents include both insulin, as well as other injectable or oral anti‐diabetic agents, including GLP‐1 receptor agonists. Anti‐psychotic medications include both typical and atypical anti‐psychotic medications. Family history was defined as people in whom a first‐degree relative had a CVD event before the age of 60 years.

### Risk scores

The following CVD risk models were evaluated: (1) D:A:D risk calculator [6], (2) the Framingham Risk Score (FRS), (3) the FRS non‐laboratory Office score [23], (4) ASCVD Pooled Cohort Equations (ASCVD PCE) [24], (5) QRISK3 [25] and (6) the World Health Organization (WHO) laboratory and (7) non‐laboratory models [26].

Risk scores necessitating laboratory results, in this case lipid measurements, included the reduced version of the D:A:D model, which is the only model evaluated that has been specifically developed for use in people living with HIV and incorporates HIV‐specific variables. Other laboratory models evaluated included the FRS and ASCVD PCE, both developed in the United States; QRISK3, a UK‐based algorithm and (5) the WHO laboratory model with region‐specific calibration.

Non‐laboratory risk scores, which rely on easily obtainable clinical information without requiring blood testing facilities and results, included the office‐based FRS and the WHO non‐laboratory model.

### Statistical analyses

Models were scaled for 5‐year CVD risk estimates for comparison with the 5‐year outcome data collected in this study, where appropriate. The D:A:D 5‐year risk calculator remained unmodified; the ASCVD PCE 5‐year equation validated in a contemporary US population was used [27]; the recalibrated WHO 5‐year equation was attained through communication with the original study author [28]; QRISK3, as well as the FRS 10‐year model equations, was rescaled to generate 5‐year predictions under a constant hazard assumption.

To assess the performance of the models, risk predictions were compared with the proportion of individuals who experienced a first‐time MACE within 5 years under each outcome definition using two complementary measures, discrimination and calibration. Discrimination refers to how well a model distinguishes between individuals who did and did not go on to experience a MACE during follow‐up. Discrimination for each risk score was evaluated using the C‐statistic, where a value of 0.5 suggests performance no better than chance, and a value of 10 indicates perfect separation of higher and lower risk individuals. Calibration assesses how closely the model's predicted probabilities align with the actual observed rates. The calibration of each risk equation was assessed by (i) calculating the ratio of observed‐to‐expected (O:E) events, calibration‐in‐the‐large (CITL) and the calibration slope and (ii) graphically comparing observed versus predicted events using calibration plots. Grouping was done by deciles of predicted risk for each equation, and pooled groups above and below the commonly used threshold for initiating primary prevention of 5% over 5 years (scaled from 10% over 10 years). Statistical analyses were performed using R Statistical Software [29] and Stata 18 [30].

### Missing data

The 5‐year predicted CVD risk scores were used in the primary analyses and were conducted as complete case analyses (CCA), excluding participants with missing baseline data required to calculate the risk score. In a secondary analysis, missing data were assumed to be Missing At Random (MAR), and risk scores were recalculated after imputing missing covariate data using multiple imputation by chained equations (MICE) [31]. All missing variables were continuous and were imputed using Predictive Mean Matching (PMM) to preserve observed distributions and accommodate non‐normality [32].

Variables requiring imputation included demographics (age, Townsend deprivation score), HIV markers (CD4 count, viral load, time since diagnosis, protease inhibitor duration), clinical measurements (body mass index, blood pressure), and laboratory values (lipid profiles). Complete variables served as predictors only. The composite cardiovascular outcome was included as a predictor but not imputed.

Twenty‐five imputations were performed with 25 iterations each to ensure convergence. The performance metrics from all datasets were then pooled using Rubin's rules that account for uncertainty from both the imputation process and model performance estimation [33].

### Sample size calculation

A feasibility study involving 245 PLWH attending a single site, Guy's and St Thomas Hospitals in London, was carried out in 2018, as part of a sub‐analysis of a previous observational cohort study of PLWH at risk of developing diabetes, the STOP Diabetes dataset (UK HRA reference 13/LO/1543) [34]. There were 9 MACE in 914.9 person‐years of follow‐up. To externally validate a prognostic model, it is recommended to have a target of 100 MACES [35]. By extrapolation and assuming the same event rate in the larger sample, 10 165 person‐years follow‐up is required for 100 events. This was estimated to involve 2100 PLWH followed up over an average of 5 years. Losses to follow up and incomplete data from a retrospective review of clinical data were considered in the above sample size calculation.

## RESULTS

### Baseline cohort demographics, characteristics, and co‐morbidities

Records from 2582 people across seven HIV clinics in England were retrospectively reviewed. Three transgender individuals were present in the overall dataset but were not included in the CCA due to the lack of data validating existing CVD risk equations in this population. A summary of the demographic details is presented in Table 1. From the CCA, the median age was 48 years (IQR 44–53 years). Half of the participants (1291/2582, 50%) were from minoritized ethnicities, with 955/2582 (37%) from Black African ethnicities. There were 745/2582 (28.9%) female participants, and 685/2582 (26.5%) reported past or current smoking use. A small proportion (356/2582, 13.8%) were taking statins at the time of the initial visit.

**TABLE 1:** Baseline characteristics of the cohort.

Predictors	Overall (*n* = 2585)	Missing (%)	CCA (*n* = 2582)	MI (*n* = 2582)
Demographics				
Median age in years [IQR]	48.0 [44.0, 53.0]	0.5	48.0 [44.0–53.0]	48.0 [44.0–53.0]
Gender				
Female	745 (28.8%)	0	745 (28.9%)	745 (28.9%)
Male	1837 (71.1%)		1837 (71.1%)	1837 (71.1%)
Transgender female^a^	2 (0.1%)			
Transgender male^a^	1 (0.04%)			
Ethnicity		0		
Black African	955 (36.9%)		955 (37%)	955 (37%)
Black Caribbean	136 (5.3%)		135 (5.2%)	135 (5.2%)
Chinese	16 (0.6%)		16 (0.6%)	16 (0.6%)
Indian	15 (0.6%)		15 (0.6%)	15 (0.6%)
Mixed ethnicity	44 (1.7%)		43 (1.7%)	43 (1.7%)
Other Asian	21 (0.8%)		21 (0.8%)	21 (0.8%)
Pakistani	3 (0.1%)		3 (0.1%)	3 (0.1%)
White or not stated	1292 (50.0%)		1291 (50.0%)	1291 (50.0%)
Any other ethnic group	103 (4.0%)		103 (4.0%)	103 (4.0%)
Median Townsend deprivation score [IQR]	4.97 [2.31, 6.72]	5.3	4.97 [2.31–6.72]	4.96 [2.31–6.72]
Cardiovascular risk factors				
Median BMI [IQR]	26.20 [23.59, 29.71]	25	26.2 [23.6–29.7]	26.2 [23.6–29.7]
Smoking status				
Non‐smoker	1898 (73.4%)	0	1897 (73.5%)	1897 (73.5%)
Ex‐smoker	215 (8.1%)		215 (8.3%)	215 (8.3%)
Heavy smoker (≥20/day)	76 (2.9%)		76 (2.9%)	76 (2.9%)
Moderate smoker (10–19/day)	252 (9.7%)		251 (9.7%)	251 (9.7%)
Light smoker (<10/day)	144 (5.6%)		143 (5.5%)	143 (5.5%)
Median total cholesterol (mmol/L) [IQR]	4.90 [4.20, 5.60]	5.2	4.90 [4.21–5.60]	4.90 [4.21–5.60]
Median HDL cholesterol (mmol/L) [IQR]	1.40 [1.13, 1.73]	7.1	1.40 [1.14–1.73]	1.40 [1.12–1.72]
Median triglycerides (mmol/L) [IQR]	1.36 [0.96, 1.99]	7.9	1.36 [0.96–1.99]	1.36 [0.95–2.00]
Median systolic blood pressure (mmHg) [IQR]	130.00 [120.00, 141.00]	23.8	130 [120–141]	130 [120–141]
Median diastolic blood pressure (mmHg) [IQR]	81.00 [75.00, 90.00]	24	81 [75–90]	81 [75–90]
Family history of CVD	157 (6.1%)	0	157 (6.1%)	157 (6.1%)
Comorbidities				
Diabetes mellitus		0		
Not diabetic	2427 (93.9%)		2424 (93.9%)	2424 (93.9%)
Type 1	16 (0.6%)		16 (0.6%)	16 (0.6%)
Type 2	142 (5.5%)		142 (5.5%)	142 (5.5%)
Chronic kidney disease	200 (7.7%)	0	200 (7.7%)	200 (7.7%)
Atrial fibrillation	16 (0.6%)	0	16 (0.6%)	16 (0.6%)
Migraines	51 (2.0%)	0	51 (2.0%)	51 (2.0%)
Rheumatoid arthritis	7 (0.3%)	0	7 (0.3%)	7 (0.3%)
Systemic lupus erythematosus	3 (0.1%)	0	3 (0.1%)	3 (0.1%)
Severe mental illness	383 (14.8%)	0	383 (14.8%)	383 (14.8%)
Erectile dysfunction	173 (6.7%)	0	173 (6.7%)	173 (6.7%)
Medication use				
On anti‐hypertensive medication	393 (15.2%)	0	393 (15.2%)	393 (15.2%)
On anti‐diabetic medication	133 (5.1%)	0	133 (5.2%)	133 (5.2%)
On atypical anti‐psychotic medication	73 (2.8%)	0	73 (2.8%)	73 (2.8%)
On regular steroid medication	40 (1.5%)	0	40 (1.5%)	40 (1.5%)
On statins	356 (13.8%)	0	356 (13.8%)	356 (13.8%)
HIV factors				
Median CD4 count (cells/μL) [IQR]	587.00 [422.00, 787.00]	0.5	587 [422–787]	587 [422–787]
Median nadir CD4 count (cells/μL) [IQR]	195.00 [84.00, 304.50]	22.1	195 [84–305]	194 [81–303]
Viral load <50 copies/ml	2205 (85.3%)	0.2	2202 (85.5%)	2207 (85.5%)
Time since HIV diagnosis (years) [IQR]	10.00 [5.00, 15.00]	0.4	10.0 [5.0–15.0]	10.0 [5.0–15.0]
Median time on PI (months) [IQR]	3.00 [0.00, 74.00]	1.5	3.0 [0.0–73.0]	3.8 [0.0–74.0]
Abacavir use		0		
Current use at baseline	394 (15.2%)		394 (15.3%)	394 (15.3%)
Previous exposure	378 (14.6%)		378 (14.6%)	378 (14.6%)
Never	1813 (70.2%)		1811 (70.1%)	1811 (70.1%)

Abbreviations: BMI, body mass index; CCA, complete cohort analysis; IQR, interquartile range; MI, multiple imputation; PI, protease inhibitors.

^a^ Three transgender participants were excluded from the analysis due to the small numbers (*n* < 5) and cardiovascular risk scores have not been validated in these populations.

The median initial CD4 cell count was 587 (Interquartile range (IQR) 422, 787) cells/μL, and 2202/2582 (85.5%) had undetectable viral loads. The median time since HIV diagnosis was 10 (IQR 5, 15) years. The prevalence of co‐morbidities is reported in Table 1, with the prevalence of severe mental illnesses (14.8%), chronic kidney disease (7.7%) and erectile dysfunction (6.7%) being most frequently reported. The demographics and characteristics of the sub‐cohort who completed five‐year follow‐up or experienced a MACE or death outcome, and the sub‐cohort of people with follow‐up <5 years were comparable when compared to account for potential bias in the cohort lost to follow‐up (Table S2).

### Observed rates of major adverse cardiovascular events

Median follow‐up was 5.0 (IQR 4.6, 5.0) years or a total of 11 553 person‐years follow‐up. There were 94 unique MACEs documented in the follow up period once subsequent events for any individuals were excluded: 17 MI, 24 ICPs, 36 cerebrovascular events, 27 CVD‐related or unexplained deaths, and 9 cases of heart failure. The MACE rate was 8.1 per 1000 person‐years follow‐up. When broken down by respective cardiovascular endpoint definitions for each risk score, there was similar overlap of CVD‐free survival despite differences in outcomes included across risk scores (Figure 1).

**FIGURE 1:**
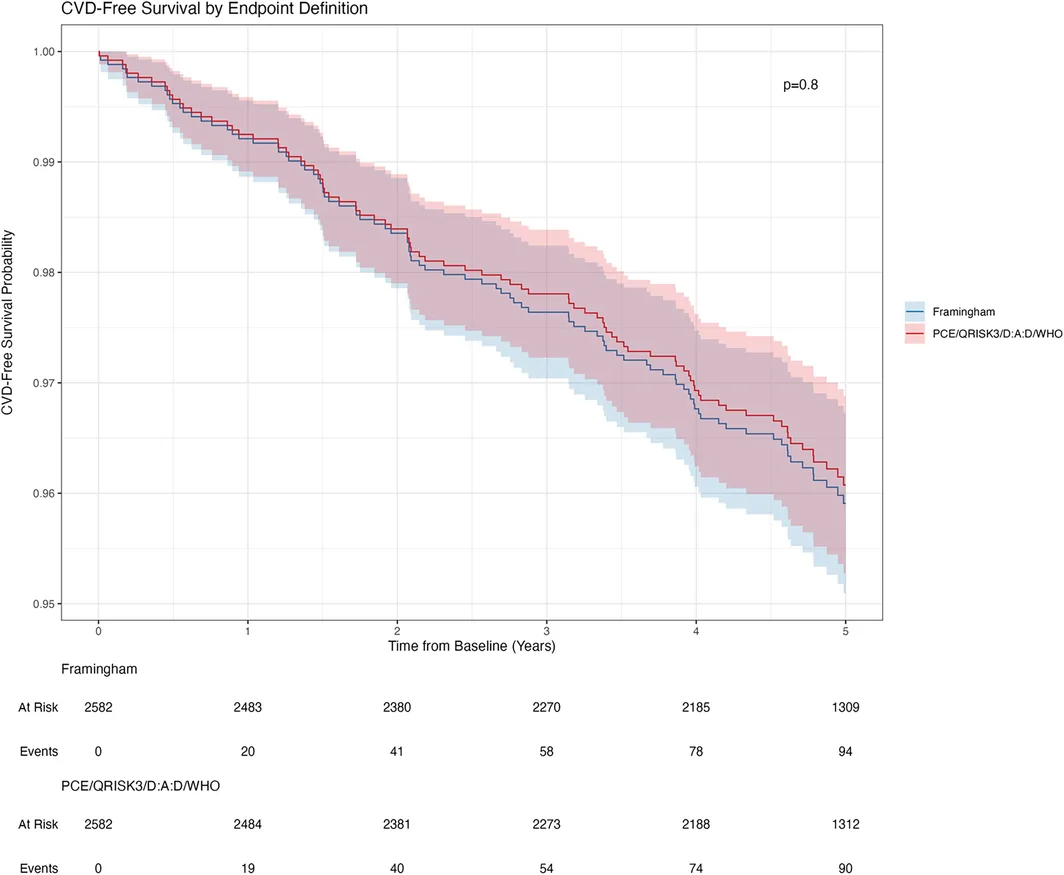
Kaplan Meier survival curves by cardiovascular endpoint definition. Kaplan–Meier (KM) survival curves showing the proportion of participants who had not experienced any major adverse CVD events (MACE) over time in years from baseline visit. Red line represents endpoints using the PCE/QRISK3/D:A:D definitions. Blue lines represent the endpoints using the Framingham equation definitions. Shaded areas represent the 95% confidence intervals for each line.

### Performance of cardiovascular risk scores

The mean and median predicted 5‐year risk scores are presented in Table 2. The FRS Office score predicted the highest median 5‐year CVD risk (4.8%, IQR 2.9%, 8.3%) and the WHO scores predicted the lowest median 5‐year CVD risk (1.5%, IQR 0.8%–2.7% for the laboratory model and 1.5%, IQR 0.8%–2.7% for the non‐laboratory model) in this cohort.

**TABLE 2:** Predicted 5‐year risk scores from complete case analysis or multiple imputation.

Risk score (*n* = 2582)	Median 5‐year risk (CCA) [IQR]	Median 5‐year risk (CCA) for statin users [IQR]	Median 5‐year risk with MI [IQR]	Median 5‐year risk with MI for statin users [IQR]
Framingham (Full)	3.6% [2.2%–6.5%]	7.1% [4.0%–11.4%]	3.6% [2.2%–6.4%]	7.1% [4.0%–11.4%]
Framingham (Office)	4.8% [2.9%–8.3%]	9.8% [5.9%–16.0%]	4.8% [2.9%–8.2%]	9.6% [5.7%–15.5%]
PCE	1.6% [0.7%–3.1%]	3.5% [1.8%–6.3%]	1.5% [0.6%–3.0%]	3.4% [1.7%–6.2%]
QRISK3	2.0% [1.1%–4.1%]	4.9% [2.6%–8.5%]	2.1% [1.1%–4.2%]	5.0% [2.7%–8.8%]
D:A:D (Reduced)	1.9% [1.1%–3.2%]	3.2% [2.1%–5.4%]	1.8% [1.1%–3.2%]	3.2% [2.1%–5.4%]
WHO CVD (Lab)	1.5% [0.8%–2.7%]	2.8% [1.6%–5.0%]	1.4% [0.8%–2.6%]	2.9% [1.6%–4.9%]
WHO CVD (Non‐Lab)	1.5% [0.9%–2.7%]	2.7% [1.5%–4.6%]	1.5% [0.8%–2.7%]	2.7% [1.5%–4.6%]

Abbreviations: CCA, complete cohort analysis; CVD, cardiovascular disease; D:A:D, data collection on Adverse events of anti‐HIV; IQR, interquartile range; MI, multiple imputation; PCE, pooled cohort equation; WHO, World Health Organization.

All seven scores demonstrated moderate discrimination performances based on the C‐statistic, ranging from 0.696 (95% CI 0.693, 0.698) for the WHO laboratory equation to 0.739 (95% CI 0.737, 0.740) for QRISK3 (Table 3). The QRISK3 equation appeared to be most closely calibrated to the study population (O:E ratio 1.169, 95% confidence interval (CI) 0.950, 1.439) with mean CITL of 0.156 (95% CI −0.052, 0.364) and slope 0.897 (0.682, 1.113). The FRS Office score predicted nearly twice as many events as observed (O:E ratio 0.542, 95% CI 0.418, 0.702), followed by the FRS full equation (O:E ratio 0.773, 95% CI 0.606, 0.985). The D:A:D (O:E ratio 1.468, 95% CI 1.145, 1.883), PCE (O:E ratio 1.572, 95% CI 1.226, 2.017), WHO laboratory (O:E ratio 1.82, 95% CI 1.424, 2.325) and WHO non‐laboratory (O:E ratio 1.599, 95% CI 1.224, 2.087) equations underpredicted MACE as observed. Details of the calibration statistics for each equation are summarized in Table 3.

**TABLE 3:** External validation metrics for 5‐year risk scores from complete case analysis.

Model	Total (*n*)	Events (*n*)	O:E ratio [95% CI]	CITL [95% CI]	Slope [95% CI]	C‐Index [95% CI]
FRS Full	1811	65	0.773 [0.606–0.985]	−0.258 [−0.501 to −0.015]	0.956 [0.662–1.251]	0.709 [0.706–0.712]
FRS Office	1741	57	0.542 [0.418–0.702]	−0.613 [−0.872 to −0.353]	0.99 [0.679–1.3]	0.721 [0.718–0.724]
PCE	1811	62	1.572 [1.226–2.017]	0.453 [0.204–0.701]	0.765 [0.511–1.018]	0.714 [0.712–0.717]
QRISK3	2568	89	1.169 [0.95–1.439]	0.156 [−0.052–0.364]	0.897 [0.682–1.113]	0.739 [0.737–0.74]
D:A:D	1811	62	1.468 [1.145–1.883]	0.384 [0.135–0.633]	1.085 [0.773–1.397]	0.728 [0.725–0.73]
WHO CVD (Lab)	1859	64	1.82 [1.424–2.325]	0.599 [0.354–0.844]	0.847 [0.564–1.131]	0.696 [0.693–0.698]
WHO CVD (Non‐Lab)	1741	54	1.599 [1.224–2.087]	0.469 [0.202–0.736]	0.937 [0.619–1.254]	0.706 [0.703–0.709]

Abbreviations: CI, confidence interval; CITL, calibration in the large metric; CVD, cardiovascular disease; D:A:D, data collection on Adverse events of anti‐HIV; FRS, Framingham risk score; O:E, observed to expected; PCE, pooled cohort equation; WHO, World Health Organization.

In the graphical evaluation of the different risk equations, the PCE and QRISK3 deciles most closely followed the perfect calibration line (Figure 2a). The Framingham office score generally overpredicted risk, especially at the highest decile. The D:A:D and WHO scores tended to underpredict risk at the highest decile. When grouped by treatment thresholds, D:A:D and PCE underprediction may result in a proportion of PLWH who are miscategorized as lower priority categories for primary CVD prevention despite observed MACE rates exceeding the treatment threshold.

**FIGURE 2:**
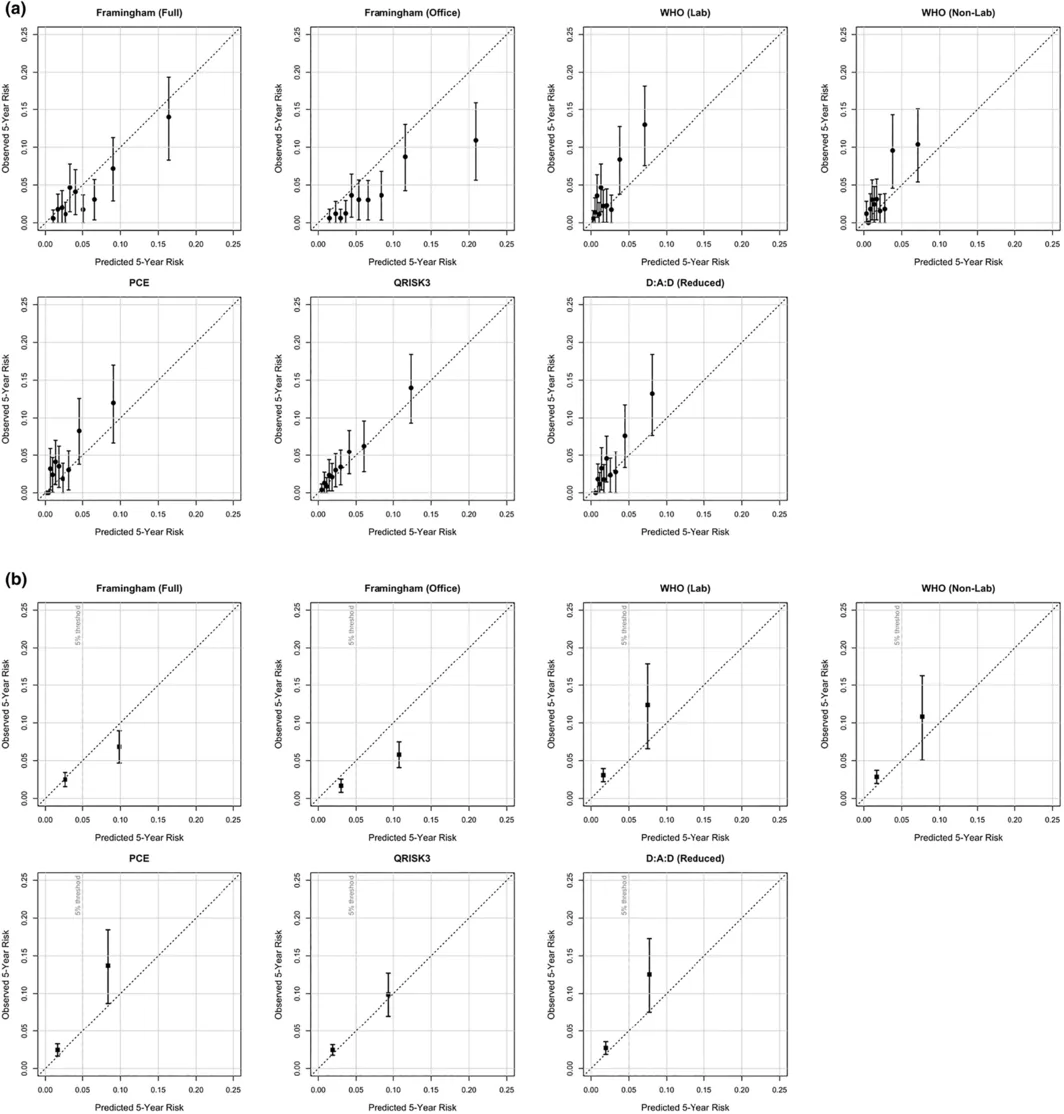
Five‐year observed CVD vs predicted probability using cardiovascular risk prediction models by decile (a) and risk category (b). Circles represent observed risk by decile; squares represent observed risk by treatment threshold (<5% vs. ≥5% 5‐year CVD risk). Error bars represent 95% confidence intervals. Dotted diagonal lines represent perfect calibration. Vertical dashed line denotes 5% treatment threshold.

### Multiple imputation

Table 2 compares the median 5‐year predicted risk using the different risk equations after imputation, with minimal difference using the imputed dataset. Calibration results and graphs using the imputed dataset were consistent with the non‐imputed results (Table S3, Figure S1).

### Sub‐analysis of individuals on statin therapy

Of the 356 people who were taking statins for primary CVD prevention at baseline, 27 (7.6%) experienced MACEs during the follow‐up period, at a rate of 16.6 per 1000 person‐years follow‐up. The median predicted CVD risk in this population was higher compared to the general population, regardless of the risk score used or multiple imputation (Table 2). Due to the small number of events, only descriptive statistics are presented for this subgroup.

## DISCUSSION

Using routinely collected data from a multi‐centre cohort of PLWH regularly attending HIV clinics in England, we demonstrate that all seven CVD risk scores showed moderate discrimination and varying calibration compared to observed MACE rates. The QRISK3, using data from national general practice databases to validate predicted CVD risk, appeared to be most closely calibrated to the CVD outcomes of a UK population of people living with well‐controlled HIV attending clinics without the need for additional calibration. Other scores may require additional local calibration to improve accuracy, particularly those that may miscategorize people who have a risk of MACE above the treatment threshold for prioritizing primary CVD prevention strategies.

This study is the first national validation of CVD risk scores in a population of PLWH in England using routinely collected clinical data. The findings add to the growing literature around the use of CVD risk prediction models in PLWH. The PCE and D:A:D equations have been previously shown to underestimate CVD risk for subgroups of PLWH in high‐income countries in a number of studies [36, 37, 38], and the WHO score was also shown to underpredict in our cohort. By 2014, there was high coverage (91%) of treatment of people who were diagnosed with HIV in England, and 95% had undetectable viral loads [39], rising to 98% treatment coverage and 98% undetectable viral loads by 2024 [40]. Compared to the previous studies, the ECARTH cohort had a relatively high ART coverage in individuals with known HIV, who were engaged in clinical care. In a cohort of PLWH with low‐moderate CVD risk in the REPRIEVE study, who were not started on statin therapy, the PCE and D:A:D were also found to overpredict cardiovascular risk in PLWH from low and middle‐income countries [37]. The use of the non‐laboratory scores, which have been suggested for low and middle‐income settings where laboratory results may not be available, may have to be used with caution, as these findings suggest over‐ or underprediction of CVD risk may be amplified with their use.

The QRISK3 [25] is the third iteration of the QRISK CVD Cox proportional hazard models that predict time to CVD events and has been externally validated independently across different UK populations [41, 42] and recommended for use in UK health guidelines. HIV has not been found to meet the model inclusion criteria in each iteration, even the most recently introduced version of QRISK‐ QR4 [43]. However, concerns around missing data, a small proportion of people diagnosed with HIV in the cohort, and younger age with shorter follow‐up in these cohorts may underestimate long‐term CVD associations [25]. Our findings address these limitations of the original QRISK3 study through the inclusion of a cohort of people with known HIV diagnoses, aged 40 years and over, regularly attending HIV clinics in the UK, demonstrating similar observed MACE rates compared to the predicted events with little calibration required. The performance of QRISK3 in our cohort, despite the absence of HIV‐related variables as covariates, may be explained by the fact that the cardiovascular effects of HIV are largely mediated through traditional risk factors, which are comprehensively captured in the QRISK3 algorithm.

Other strengths of the study include a diverse population with half the cohort from minoritized ethnicities, a large proportion of women living with HIV, and the relative completeness of the data with multiple imputation analyses to evaluate the impact of the remaining missing fields. The use of routinely collected clinical data provides results reflecting the real‐world performance of these cardiovascular risk scores. Following the results of the REPRIEVE study, which demonstrated the efficacy of statins for CVD primary prevention for PLWH aged above 40 regardless of predicted CVD risk [16], an increasing number of HIV management guidelines now recommend statins for CVD primary prevention for PLWH [17, 18]. However, evaluating the accuracy of CVD risk prediction tools in a real‐world clinical setting remains highly pertinent to prioritize the people at highest risk of MACE for primary CVD prevention strategies due to clinical resource limitations [17].

There were limitations to this study. The study only included people who were engaged in healthcare, and may not reflect those not accessing HIV care in England and who may have higher levels of CVD risk. As previously discussed, ART uptake by people living with HIV is high, with 98% of people diagnosed with HIV on ART and 98% of those undetectable [40], suggesting that the remaining population of people not accessing HIV care in England is low and decreasing. Another limitation is the low number of MACE, which meant further sub‐grouping, e.g., by ethnicities, gender or statin use, was not possible beyond descriptive analyses.

In conclusion, using real‐world clinical data from people with HIV engaging in care in England, we found CVD risk scores performed moderately well overall, with the best performance from risk scores validated using UK primary care datasets. Future models may benefit from incorporating a broader range of variables beyond traditional CVD risk factors, such as markers of choring inflammation, immune activation, HIV or ART‐specific factors, and potentially socioeconomic or behavioural determinants. The best performing model was the one developed in the context of the UK‐based ECARTH population, emphasizing the need to calibrate each CVD risk score to the local population it is used for. Caution should be advised in using non‐laboratory‐based scores where validation studies may be limited in the settings where they are commonly used due to the paucity of long‐term cohort data with accurate CVD outcomes.

## AUTHOR CONTRIBUTIONS

MJL, CS, RK, ASW conceived and designed the study. AD provided data for the sample size calculations. NN, AS are site research managers. MJL, ZA, AD, ED, MG, MH, MJ, FL, GM, JM, CP, IR, RS, ET, JJT, RK are site investigators responsible for data collection. CS, AM, RS, TRF, JD are responsible for the data analysis. MJL, AM, CS, RS, TRF, JD, RK, ASW contributed to the first manuscript draft. All authors reviewed and contributed to the final manuscript.

## FUNDING INFORMATION

This study is funded by the Guy's and St Thomas Charity Multiple Long Term Conditions Fund (EIC180702). MJL was funded by the UK Medical Research Council Clinical Research Training Fellowship (MR/W024454/1). TRF receives funding from the National Institute for Health and Care Research (NIHR) HealthTech Research Centre for Community Healthcare at Oxford Health NHS Foundation Trust (NIHR205287) and the NIHR Applied Research Collaboration Oxford and Thames Valley at Oxford Health NHS Foundation Trust (NIHR200172). JD is funded by the NIHR (CL‐2022–13–005). The views expressed are those of the authors and not necessarily those of the NHS, the NIHR or the Department of Health and Social Care.

## CONFLICT OF INTEREST STATEMENT

The authors declare no conflicts of interest.

## ETHICS STATEMENT

The study was approved by the UK Health Research Authority (UK HRA) (REC reference 20/HRA/6306) and deemed exempt from Research Ethics Committee review due to the retrospective nature of the study and review of previously collected, non‐identifiable routine clinical information.

## Supporting information


**Table S1.** List of participating clinics and participant demographics by clinics.
**Table S2.** Cohort characteristics of the cohort who completed 5‐year follow‐up or experienced a MACE or death outcome against those with <5 years follow‐up.
**Table S3.** External validation metrics for 5‐year risk scores from multiple imputation.
**Figure S1.** Post‐multiple imputation 5‐year observed CVD vs predicted probability using cardiovascular risk prediction models by decile (A) and risk category (B) of one representative imputed dataset. Circles represent observed risk by decile; squares represent observed risk by treatment threshold (<5% vs. ≥5% 5‐year CVD risk). Error bars represent 95% confidence intervals. Dotted diagonal lines represent perfect calibration. The vertical dashed line denotes the 5% treatment threshold.

## Data Availability

Pseudonymized study data will be available to other researchers on request with approval from the trial steering committee and appropriate research ethics committee approval.
