# Supplementary material for: A multicentre evaluation of cardiovascular risk prediction tools in people living with HIV across England (ECARTH study)
Source: HIV Med. 2026 Jul 20;27(10):1629–41. doi: 10.1111/hiv.70282 (PMC13631688; doi:10.1111/hiv.70282)
Supplement: Supplementary file 1 [file HIV-27-1629-s001.docx]

**Supplementary table 1. List of participating clinics and participant demographics by clinics**

|  | **Total cohort** | **Guy’s and St Thomas Hospital NHS Foundation Trust** | **West Middlesex Hospital, Chelsea and Westminster Hospital NHS Foundation Trust** | **Imperial College Healthcare NHS Trust** | **Homerton University Hospital NHS Foundation Trust** | **North Bristol NHS Trust** | **Leeds Teaching Hospitals NHS Trust** | **Royal Free London NHS Foundation Trust** |
| --- | --- | --- | --- | --- | --- | --- | --- | --- |
| **Total number of patients in cohort** | 2585 | 2041 | 50 | 98 | 96 | 100 | 100 | 100 |
| **Median Age in years (IQR)** | 48 (44, 53) | 48 (44,53) | 50 (44, 55) | 49 (45, 54) | 55 (50, 62) | 48 (43, 54) | 47 (42, 52) | 49 (44, 54) |
| **Ethnicity** |  |  |  |  |  |  |  |  |
| - **White** | 1292 (50.0%) | 1025 (50.1%) | 18 (36.0%) | 54 (55.1%) | 26 (27.1%) | 63 (63.0%) | 46 (46.0%) | 60 (60.0%) |
| - **Non-white** | 1293 (50.0%) | 1016 (49.8%) | 32 (64.0%) | 44 (44.9%) | 70 (72.9%) | 37 (37.0%) | 54 (54.0%) | 40 (40.0%) |
| **Gender** |  |  |  |  |  |  |  |  |
| - **Male** | 1837 (71.1%) | 1482 (72.6%) | 28 (56.0%) | 74 (75.5%) | 46 (47.9%) | 69 (69.0%) | 62 (62.0%) | 76 (76.0%) |
| - **Female** | 745 (28.8%) | 556 (27.2%) | 22 (44.0%) | 24 (24.5%) | 50 (52.1%) | 31 (31.0%) | 38 (38.0%) | 24 (24.0%) |
| - **Trans/non-binary** | 3 (0.0%) | 3 (0.0%) | 0 (0.0%) | 0 (0.0%) | 0 (0.0%) | 0 (0.0%) | 0 (0.0%) | 0 (0.0%) |

IQR: Interquartile Range

**Supplementary table 2. Cohort characteristics of the cohort who completed five-years follow up or experienced a MACE or death outcome against those with less than five years follow up**

| **Predictors** | **Event, death or 5 years follow-up (n=1446)** | **Less than 5 years follow-up (n=1136)** |
| --- | --- | --- |
| **Demographics**  Median age in years [IQR]  Gender  Female  Male  Ethnicity  Black African  Black Caribbean  Chinese  Indian  Mixed ethnicity  Other Asian  Pakistani  White or not stated  Any other ethnic group  Median Townsend deprivation score [IQR]  **Cardiovascular risk factors**  Median BMI [IQR]  Smoking status  Non-smoker  Ex-smoker  Current smoker  Median total cholesterol (mmol/L) [IQR]  Median HDL cholesterol (mmol/L) [IQR]  Median triglycerides (mmol/L) [IQR]  Median systolic blood pressure (mmHg) [IQR]  Median diastolic blood pressure (mmHg) [IQR]  Family history of CVD  **Comorbidities**  Diabetes mellitus  Not diabetic  Type 1  Type 2  Chronic kidney disease  Atrial fibrillation  Migraines  Rheumatoid arthritis  Systemic lupus erythematosus  Severe mental illness  Erectile dysfunction  **Medication use**  On antihypertensive medication  On antidiabetic medication  On atypical antipsychotic medication  On regular steroid medication  On statins  **HIV factors**  Median CD4 count (cells/μL) [IQR]  Median nadir CD4 count (cells/μL) [IQR]  Viral load <50 copies/ml  Time since HIV diagnosis (years) [IQR]  Median time on PI (months) [IQR]  Abacavir use  Current use at baseline  Previous exposure  Never | 48.0 [44.0, 54.0]  436 (30.2%)  1010 (69.8%)  564 (39.0%)  91 (6.3%)  10 (0.7%)  8 (0.6%)  20 (1.4%)  14 (1.0%)  1 (0.1%)  674 (46.6%)  64 (4.4%)  5.32 [2.91, 6.84]  26.30 [23.54, 29.91]  1055 (73.0%)  133 (9.2%)  258 (17.8%)  4.90 [4.30, 5.60]  1.40 [1.16, 1.75]  1.34 [0.96, 1.94]  130.00 [120.00, 142.00]  81.00 [75.00, 90.00]  85 (5.9%)  1335 (92.4%)  10 (0.7%)  101 (7.0%)  128 (8.9%)  11 (0.8%)  32 (2.2%)  3 (0.2%)  2 (0.1%)  248 (17.2%)  110 (7.6%)  241 (16.7%)  95 (6.6%)  47 (3.3%)  29 (2.0%)  211 (14.6%)  583.00 [417.75, 787.00]  188.00 [78.00, 290.25]  1240 (86.0%)  10.00 [5.00, 15.00]  6.00 [0.00, 79.00]  211 (14.6%)  224 (15.5%)  1011 (69.9%) | 48.0 [43.0-53.0]  309 (27.2%)  827 (72.8%)  391 (34.4%)  44 (3.9%)  6 (0.5%)  7 (0.6%)  23 (2.0%)  7 (0.6%)  2 (0.2%)  617 (54.3%)  39 (3.4%)  4.30 [1.69, 6.44]  26.01 [23.74, 29.32]  212 (18.7%)  82 (7.2%)  212 (18.7%)  4.90 [4.20, 5.70]  1.40 [1.10, 1.72]  1.38 [0.95, 2.05]  130.00 [120.00, 141.00]  81.00 [75.00, 89.00]  72 (6.3%)  1089 (95.9%)  6 (0.5%)  41 (3.6%)  72 (6.3%)  5 (0.4%)  19 (1.7%)  4 (0.4%)  1 (0.1%)  135 (11.9%)  63 (5.5%)  152 (13.4%)  38 (3.3%)  26 (2.3%)  11 (1.0%)  145 (12.8%)  588.50 [427.50, 786.25]  206.00 [93.00, 320.00]  962 (84.8%)  10.00 [5.25, 15.00]  0.00 [0.00, 68.00]  183 (16.1%)  153 (13.5%)  800 (70.4%) |

**Supplementary table 3. External validation metrices for 5 year risk scores from multiple imputation**

| **Model (n = 2582)** | **Events (n)** | **O:E**  **[95% CI]** | **CITL**  **[95% CI]** | **Slope**  **[95% CI]** | **C-Index**  **[95% CI]** |
| --- | --- | --- | --- | --- | --- |
| FRS (Full)  FRS (Office)  PCE  QRISK3  D:A:D (Reduced)  WHO CVD (Lab)  WHO CVD (Non-Lab) | 94  94  90  90  90  90  90 | 0.795 [0.649-0.974]  0.613 [0.501-0.751]  1.629 [1.325-2.004]  1.135 [0.923-1.396]  1.521 [1.237-1.87]  1.878 [1.527-2.31]  1.816 [1.477-2.234] | -0.229 [-0.432--0.027]  -0.489 [-0.692--0.287]  0.488 [0.281-0.695]  0.127 [-0.08-0.333]  0.419 [0.212-0.626]  0.63 [0.424-0.837]  0.597 [0.39-0.804] | 0.923 [0.676-1.17]  0.912 [0.663-1.16]  0.752 [0.542-0.963]  0.898 [0.687-1.108]  1.041 [0.785-1.297]  0.864 [0.624-1.104]  0.800 [0.550-1.050] | 0.703 [0.689-0.717]  0.708 [0.693-0.723]  0.711 [0.701-0.721]  0.742 [0.733-0.75]  0.724 [0.714-0.733]  0.702 [0.691-0.714]  0.682 [0.665-0.698] |

CI: confidence interval, CITL: Calibration in the large metric, CVD: Cardiovascular disease, D:A:D: Data collection on Adverse events of anti-HIV, O:E: Observed to Expected, FRS: Framingham Risk Score, PCE: Pooled Cohort Equation; WHO: World Health Organisation

**Supplementary Figure 1.**

**A**

**B**

**Post-multiple imputation five-year observed CVD vs predicted probability using cardiovascular risk prediction models by decile (A) and risk category (B) of one representative imputed dataset.** Circles represent observed risk by decile; squares represent observed risk by treatment threshold (<5% vs ≥5% 5-year CVD risk). Error bars represent 95% confidence intervals. Dotted diagonal lines represent perfect calibration. Vertical dashed line denotes 5% treatment threshold.
